# Structure of K102 Capsular Polysaccharide from *Acinetobacter baumannii* KZ-1102 and Its Cleavage by Phage Cato Depolymerase

**DOI:** 10.3390/ijms26104727

**Published:** 2025-05-15

**Authors:** Anastasia A. Kasimova, Nikolay P. Arbatsky, Ekaterina A. Gornostal, Mikhail M. Shneider, Eugene A. Sheck, Alexander S. Shashkov, Andrey A. Shelenkov, Yulia V. Mikhailova, Ilya S. Azizov, Mikhail V. Edelstein, Andrey V. Perepelov, Anna M. Shpirt, Konstantin A. Miroshnikov, Anastasia V. Popova, Yuriy A. Knirel

**Affiliations:** 1 N. D. Zelinsky Institute of Organic Chemistry, Russian Academy of Sciences, 119991 Moscow, Russia; nastia-kasimova979797@mail.ru (A.A.K.); nikolay.arbatsky@gmail.com (N.P.A.); shash@ioc.ac.ru (A.S.S.); perepel@ioc.ac.ru (A.V.P.); asyashpirt@gmail.com (A.M.S.); yknirel@gmail.com (Y.A.K.); 2 State Research Center for Applied Microbiology and Biotechnology, 142279 Obolensk, Russia; 3 M. M. Shemyakin and Yu. A. Ovchinnikov Institute of Bioorganic Chemistry, Russian Academy of Sciences, 117997 Moscow, Russia; katiealarusse@gmail.com (E.A.G.); mikhailshneider@gmail.com (M.M.S.); kmi@bk.ru (K.A.M.); 4 Institute of Antimicrobial Chemotherapy, Smolensk State Medical University, 214019 Smolensk, Russia; evgeniy.sheck@antibiotic.ru (E.A.S.); ilya.azizov@antibiotic.ru (I.S.A.); mikhail.edelstein@antibiotic.ru (M.V.E.); 5 Central Scientific Research Institute of Epidemiology, 111123 Moscow, Russiamihailova@cmd.su (Y.V.M.)

**Keywords:** *Acinetobacter baumannii*, capsular polysaccharide, capsular polysaccharide structure, K type, phage tailspike depolymerase, depolymerization

## Abstract

*Acinetobacter baumannii* is a significant nosocomial pathogen characterized by the ability to produce a wide variety of capsular polysaccharides (CPSs). The structures of a K102-type CPS isolated from *A. baumannii* KZ-1102 and its Smith degradation product were determined by sugar analysis, 1D and 2D ^1^H NMR spectroscopy, and ^13^C NMR spectroscopy. The K102 CPS biosynthesis gene cluster (KL102) contains genes for common sugar synthesis, K unit processing, capsule export, glycosyl transfer, initiating sugar phosphate transfer, and genes that encode d-Glc*p*NAc/d-Gal*p*NAc dehydrogenase and phosphoglycerol transferase. The CPS is composed of a pentasaccharide repeating unit (K unit) consisting of a tetrasaccharide backbone including one α-d-Gal*p*, three α-d-Glc*p*NAc residues, and one residue of a β-d-Glc*p* as a side chain. The tailspike depolymerase of the specific *Obolenskvirus* phage Cato was found to cleave the α-d-Glc*p*NAc-(1→6)-α-d-Glc*p*NAc linkage in the K102 CPS to give the monomer and dimer of the K repeating unit, which were characterized by high-resolution electrospray ionization mass spectrometry as well as ^1^H and ^13^C NMR spectroscopy.

## 1. Introduction

*Acinetobacter baumannii* is a Gram negative, strictly aerobic, nonmotile, non-fermenting, catalase-positive, and oxidase-negative coccobacillus belonging to the family *Moraxellaceae* [1]. Over the past decades, *Acinetobacter baumannii* has emerged as one of the most significant opportunistic pathogens associated with a wide range of hospital-acquired infections [2,3,4]. As a representative of the «ESKAPE» group (*Enterococcus faecium*, *Staphylococcus aureus*, *Klebsiella pneumoniae*, *Acinetobacter baumannii*, *Pseudomonas aeruginosa,* and *Enterobacter* species), *A. baumannii* is characterized by resistance to multiple classes of antibiotics and antibacterial drugs [5,6,7]. In this regard, the study of the *A. baumannii* population structure and the search for alternative antibacterial agents effective against this microorganism are becoming subjects of particular interest.

The majority of *A. baumannii* strains produce capsular polysaccharides (CPSs), which form a thick protective layer around bacterial cells and are the key surface components contributing to bacterial cell survival, virulence, evasion of the host immune system, and participation in biofilm formation [3,8,9,10,11]. Currently, more than 240 CPS biosynthesis gene clusters (KL) have been bioinformatically predicted in the *A. baumannii* genomic sequences deposited in the NCBI database [12]. The CPS structures of more than 64 *A. baumannii* capsular types (K types) have been biochemically confirmed and established [13]. CPSs are high-molecular-weight polymers consisting of repeating oligosaccharide units (K units) that differ from each other in terms of sugar composition, linkages between sugars and K units, and the decoration of K units with different groups or other moieties [14]. The K units of the *A. baumannii* CPSs contain from 3 to 8 monosaccharides, most of which are usually widespread and common, such as galactose, glucose, glucosamine, and glucuronic acid, but there are also rare residues, such as d-alanine, d-ribose, diaminoquinavosamine, and mannosaminuronic acid [13]. Considering the variability of capsular polysaccharides produced within a population of *A. baumannii*, determining whether an isolate belongs to a certain K type is critical for the development of vaccines and preparations based on specific phages or phage-derived enzymes.

In this study, the previously undescribed K102 CPS structure from *A. baumannii* isolate KZ-1102 and its correlation with the capsular biosynthesis gene cluster KL102 were determined. *A. baumannii* KZ-1102 is a bacterial host for the specific phage Cato, the biological properties and genomic organization of which were previously characterized [15]. Like the other members of the genus *Obolenskvirus*, this phage has a complex adsorption apparatus made of tail fiber protein and tailspike depolymerase, which are responsible for degrading the CPS of a certain structure [15,16]. The structures of oligosaccharide products derived after the depolymerization of K102 CPS by the specific depolymerase Cato_gp43 encoded in the phage genome were also examined.

## 2. Results

### 2.1. Characteristics of the A. baumannii KZ-1102 Isolate

*A. baumannii* KZ-1102 was originally isolated from a sputum specimen of a 1-year-old male patient with nosocomial pneumonia in April 2017 in Astana, Kazakhstan. The isolate was tested for susceptibility to antibiotics using a reference broth-microdilution susceptibility testing method according to ISO 20776-1:2019 [17]. *A. baumannii* KZ-1102 was found to be fully susceptible to imipenem, meropenem, amikacin, gentamicin, tobramycin, colistin, and trimethoprim-sulfamethoxazole, according to the EUCAST clinical breakpoints [18]. By multilocus sequence typing (MLST) analysis, KZ-1102 was assigned to sequence type (ST) 1560 by the Institut Pasteur MLST scheme and ST 2214 by the University of Oxford MLST scheme [19,20].

### 2.2. Characterization of the KL102 CPS Biosynthesis Gene Cluster

**Figure 1:**
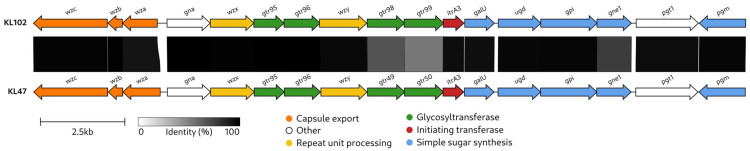
Comparison of KL102 (MK399429; the region corresponds to the KL102: 3274–24,356 bp) and KL47 (MN166193). The arrows indicate genes in the transcription direction. The maps were created using Clinker [23]. The sequence similarity percentage is indicated by the color intensity shown in the legend below. The scale bar and color scheme are also shown below.

The CPS biosynthesis gene cluster identified in the KZ-1102 genome sequence was assigned to KL102 (GenBank accession number: MK399429; the region corresponding to KL102 is as follows: 3274–24,356 base pairs (bp), between genes *wzc* and *pgm*). KL shares 99% coverage and 97.49% nucleotide sequence identity with KL102 from MSHR_200 (GenBank accession number: MK370021), which was isolated in Australia [21]. KL102 from *A. baumannii* KZ-1102 also shares a fairly high level of similarity with the KL47 (MN166193; the coverage obtained to an E-value of 0 was 95% with an identity of 97.63%) identified in *A. baumannii* NIPH601 [22] (Figure 1). Both clusters contain genes for common sugar synthesis (*galU*, *ugd*, *gpi*, *gne1*, and *pgm*), K unit processing (*wzx*, *wzy*), capsule export (*wza*, *wzb*, and *wzc*), glycosyl transfer (*gtr*), initiating sugar phosphate transfer (*itr*), and genes *gna* and *pgt1* that encode d-Glc*p*NAc/d-Gal*p*NAc dehydrogenase and phosphoglycerol transferase, respectively. KL102 and KL47 differ from each other in a region containing the glycosyltransferase genes *gtr98* and *gtr99* (in KL102) and *gtr49* and *gtr50* (in KL47). The absence of a module for the synthesis of complex sugars suggests that only simple sugars (Glc*p*, Gal*p*, Glc*p*NAc, Gal*p*NAc, and/or GlcA) are present in the K102 CPS. Notably, Wzy_K102_ (GenBank accession number: QBM04763) and Wzy_K47_ (QHB12947) are almost identical (the coverage obtained to an E-value of 0 was 98% with an identity of 99.12%), indicating that they form the same linkages between the K102 and K47 units.

### 2.3. Resolution of the K102 CPS Structure

The K102 CPS was isolated by phenol-water extraction [24] from *A. baumannii* KZ-1102 and purified by gel permeation chromatography (GPC). Sugar analysis of the CPS by gas–liquid chromatography (GLC) of the acetylated alditols revealed the presence of d-GlcN, d-Gal, and d-Glc in the ratios ~1.8:0.7:0.7, respectively.

**Figure 2:**
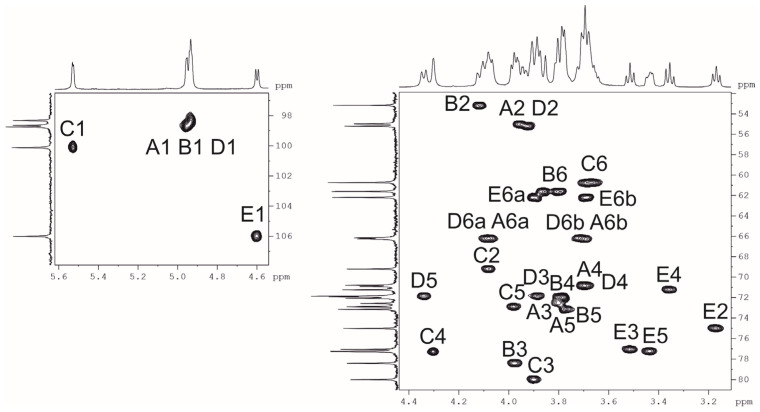
The ^1^H,^13^C HSQC NMR spectrum of the CPS *A. baumannii* KZ-1102.

Nuclear magnetic resonance (NMR) spectra showed that the sample was contaminated with non-carbohydrate compounds, but an attempt to purify it by GPC failed because of its high viscosity. Therefore, the crude preparation was heated under mild acidic conditions and fractionated by GPC on G-50 to produce a purified sample of CPS. The NMR spectra of the CPS were assigned using two-dimensional ^1^H,^1^H correlation spectroscopy (COSY), ^1^H,^1^H total correlation spectroscopy (TOCSY), ^1^H,^1^H rotational frame nuclear overhauser effect spectroscopy (ROESY), and ^1^H,^13^C heteronuclear single quantum coherence (HSQC) (Figure 2) experiments.

In the ^1^H NMR spectrum, signals of five anomeric protons were observed at δ_H_ 4.60 (β-sugar) and 4.93–5.53 (α-sugars), three characteristic signals of NCOCH_3_-groups at δ_H_ 2.06–2.07, three double signals of CH_2_OH-groups at δ_H_ 3.67–3.89, and two for substituted CH_2_OH-groups at δ_H_ 3.71 and 4.08 (Table 1). The ^13^C NMR spectrum showed that four residues have α-configurations (δ_C_ 98.3–100.1), whereas one residue has a β-configuration (at δ_C_ 106.0) [25]. The spectrum also included the signals of three COCH_3_-groups at δ_C_ 23.2–23.5, three nitrogen-bearing carbons at C2 of GlcNAc at δ_C_ 55.0–55.2, three NHCO groups at δ_C_ 175.3–175.6, three CH_2_OH-groups at δ_C_ 60.7–62.2, and two substituted CH_2_OH-groups at δ_C_ 66.2, 66.3.

Assignment of the ^1^H and ^13^C NMR spectra was performed using 2D ^1^H,^1^H (COSY, TOCSY, and ROESY) and ^1^H,^13^C (HSQC, HMBC) experiments, which revealed the spin systems for each of the five residues, all being in the pyranose form. On the basis of these data, it was concluded that the K unit of the CPS consists of three Glc*p*NAc residues, one Gal*p* residue, and one Glc*p* residue.

The COSY experiment revealed H1/H2 correlations, and their J_1,2_ constants point out the α-anomeric configuration of all three GlcNAc residues, one Gal residue (J_1,2_ < 3), and the β-configuration of a Glc residue (J_1,2_ > 7).

The HSQC spectrum showed the points of substitution for each residue due to their low-field positions of the substituted carbons [25]. The ^1^H,^1^H ROESY experiment showed correlations between the anomeric protons and protons at the linkage carbons: D1/C4, C1/B3, B1/A6, A1/D6, and E1/C3 at δ_H_ 4.96/4.31, 5.53/3.97, 4.93/3.71, 4.08, 4.94/3.71, 4.08, and 4.60/3.90, respectively.

**Figure 3:**
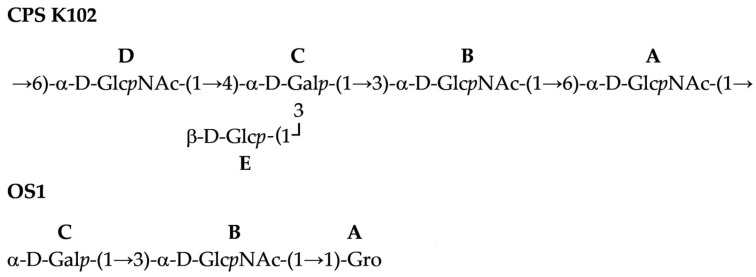
Structures of the K102 CPS of *A. baumannii* KZ-1102 and OS1 derived after Smith degradation.

Therefore, the CPS consisted of branched K units with four monosaccharide residues (A–D) in the main chain, and β-d-Glc (E) as a side residue (Figure 3). The CPS structure was confirmed by Smith degradation, which resulted in the destruction of the side residue of Glc*p* and two C6-substituted Glc*p*NAc residues in the main chain to give a disaccharide α-d-Gal*p*-(1→3)-α-d-Glc*p*NAc-(1→1)-Gro (OS1). Its structure was established by NMR spectroscopy (Table 2) as for initial CPS.

### 2.4. K102 CPS Cleavage by Specific Depolymerase Cato_gp43

**Figure 4:**
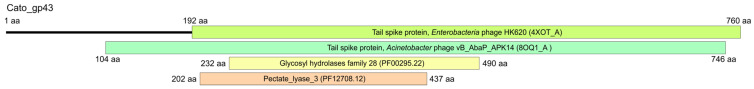
HHpred-detected similarities between Cato_gp43 and proteins from the Pfam and PDB databases.

The genome of the previously described *Obolenskvirus* phage Cato (GenBank accession number: OM471864), which infects *A. baumannii* KZ-1102 [15], as well as the genomes of the other phages of this genus, encodes only one tailspike depolymerase that determines specificity to a certain *A. baumannii* K type [16]. Recombinant Cato-derived depolymerase lacking the N-terminal domain formed an opaque halo (zone of depolymerization of K102 CPS) on the bacterial lawn of *A. baumannii* KZ-1102 [15]. According to HHpred analysis [26], the amino acid sequence of Cato_gp43 had the pectate lyase 3 (PF12708.12; E-value of 1.7 × 10^−9^) and Glyco_hydro_28 (PF00295.22; E-value of 1.2 × 10^−9^) conserved Pfam motifs and was found to be structurally similar to tailspikes of different virulent phages, including *A. baumannii* phages (Figure 4).

The BLASTp analysis revealed that the CPS-recognizing/degrading part (137–760 amino acids) of the K102-specific depolymerase Cato_gp43 (GenBank accession number: UMO77867) is similar to the depolymerase TaPaz_gp79 (QVW53860) encoded by the *A. baumannii* phage TaPaz with a determined substrate specificity toward the K47 *A. baumannii* NIPH601 [27]. This indicates that K102-specific Cato_gp43 and K47-specific TaPaz_gp79 are presumed to recognize and degrade the same linkage within the K102 and K47 CPSs.

**Figure 5:**
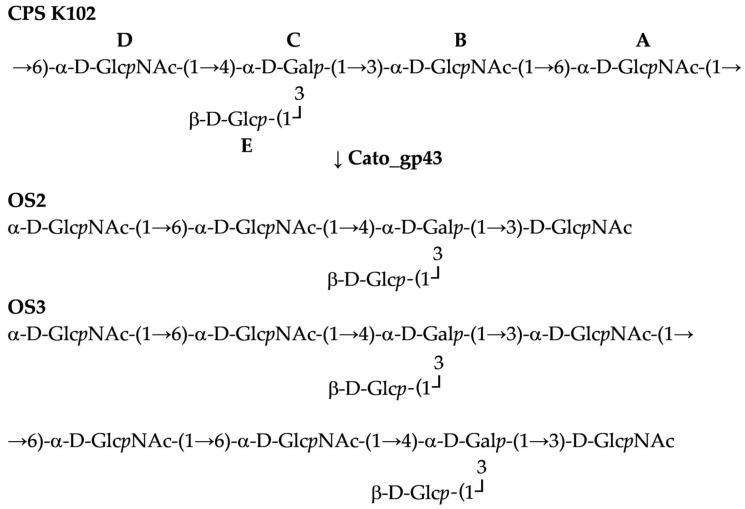
Cleavage of the K102 CPS of *A. baumannii* KZ-1102 by phage-derived depolymerase Cato_gp43.

To determine whether a precise linkage in the K102 CPS from *A. baumannii* KZ-1102 was cleaved, a fragment encoding the CPS-recognizing/degrading part of the structural depolymerase Cato_gp43 was cloned [15], purified, and then used for digestion. The recovered products were fractionated by GPC, and the resultant oligosaccharides OS2 and OS3 were studied by high-resolution electrospray ionization mass spectrometry (HR ESI MS) and found to correspond to the monomer and dimer of the K102 CPS repeating unit, respectively (Figure 5).

**Figure 6:**
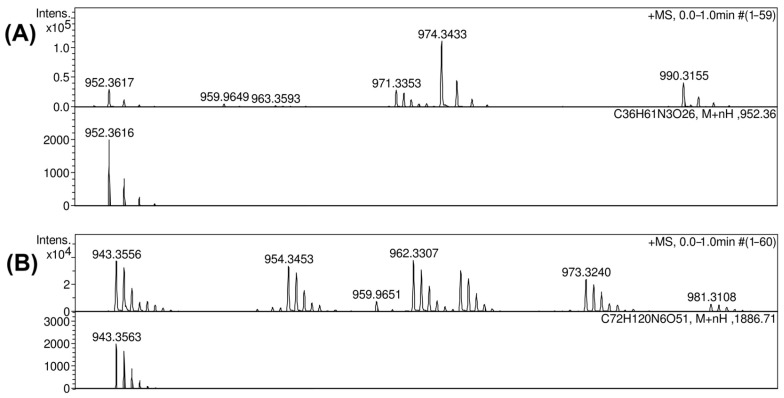
Mass spectra of the resultant oligosaccharides OS2 and OS3 obtained after cleavage of the K102 CPS by phage-derived depolymerase Cato_gp43. (**A**) Mass spectra of the OS2. (**B**) Mass spectra of the OS3.

The positive ion mode mass spectra of OS2 gave [M + H]^+^ ion, *m*/*z* 952.3617 (calcd. *m*/*z* 952.3616) (Figure 6A), and OS3 [M + 2H]^2+^ ion, *m/z* 943.3556 (calcd. *m*/*z* 943.3563) (Figure 6B).

**Figure 7:**
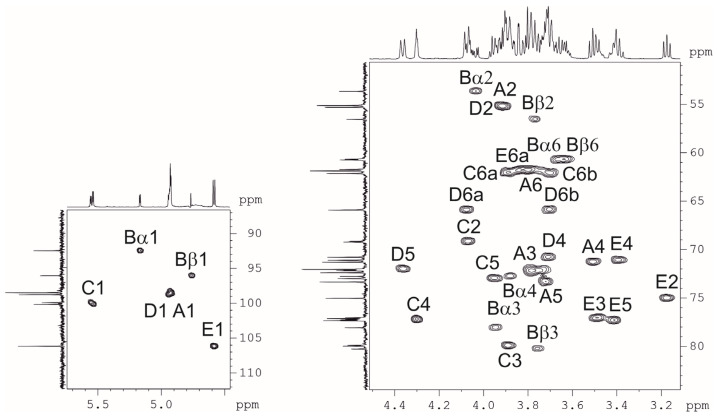
The ^1^H,^13^C HSQC NMR spectrum of OS2 derived after depolymerization of the K102 CPS of *A. baumannii* KZ-1102 by specific depolymerase Cato_gp43.

The full structure of OS2 was established by two-dimensional NMR spectroscopy including ^1^H,^1^H COSY, TOCSY, ROESY, ^1^H,^13^C HSQC (Figure 7), and HMBC experiments (Table 3). The reducing end of pentasaccharide OS2 was occupied by Glc*p*NAc, which occurs as a mixture of the α- and β-anomers. The structure of the higher decasaccharide OS3 was proposed based on the structures of CPS and OS2 using 2D ^1^H and ^13^C experiments.

These data indicate that phage depolymerase is a glycosidase that specifically cleaved the α-d-Glc*p*NAc-(1→6)-α-d-Glc*p*NAc linkage between repeating units in the K102 CPS.

## 3. Discussion

To date, CPS structures for more than 64 *A. baumannii* K types have been determined [13]. In this study, the first reported structure of K102 CPS from *A. baumannii* KZ-1102, isolated in Kazakhstan, was resolved. The CPS includes only common neutral monosaccharides (Glc*p*, Gal*p*, and Glc*p*NAc) within repeating oligosaccharide K units, which was bioinformatically confirmed by the analysis of the KL102 gene content.

**Figure 8:**
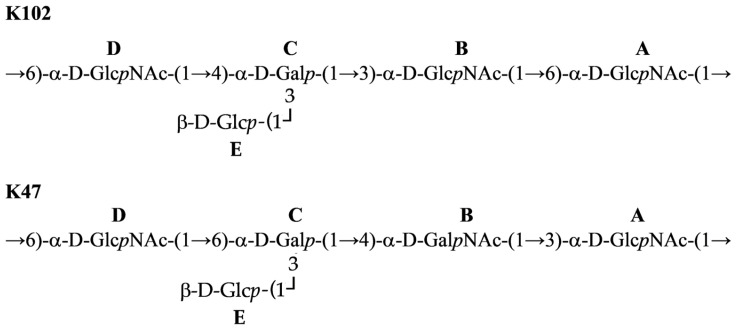
*A. baumannii* K102 and K47 CPS structures.

The KL102 identified in the *A. baumannii* KZ-1102 genome sequence shares a fairly high nucleotide identity and most gene content with KL47 identified in *A. baumannii* NIPH601 [22], differing in only a small region that includes the glycosyltransferase genes (*gtr98*, *gtr99* in KL102 and *gtr49*, *gtr50* in KL47). These genes encode glycosyltransferase enzymes that form different glycosidic linkages between sugars within repeating K units. Accordingly, the CPS structures of *A. baumannii* K102 and K47 were also similar. Both CPS are presented by a branched pentasaccharide repeating unit consisting of simple sugars (Figure 8). The structures of K102 and K47 CPSs were shown to include the same linkage (α-d-Glc*p*NAc-(1→6)-α-d-Glc*p*NAc) between the K102 and K47 units because of the shared almost identical gene *wzy* in the KL102 and KL47 gene clusters.

In this study, the structures of oligosaccharide products obtained after depolymerization of K102 CPS by the recombinant enzyme Cato_gp43 encoded in the genome of the *Obolenskvirus* phage Cato were also established. To date, the mechanisms of enzymatic activity only for two *Obolenskvirus* phage-derived tailspike depolymerases have been described—that of the K91(40)-specific AP22-derived depolymerase AP22_gp54 [28] and the K82-specific Scipio-derived depolymerase Scipio_gp39 [16]. K102-specific depolymerase, as well as previously described K82-specific depolymerase, was shown to be a specific glycosidase that cleaves the corresponding *A. baumannii* CPS by a hydrolytic mechanism. The CPS-recognizing/degrading part of Cato_gp43 shares a similarity at the amino acid level with the corresponding part of tailspike depolymerase TaPaz_gp79. Moreover, Cato_gp43 and TaPaz_gp79, both glycosidases, were found to specifically cleave the α1 → 6-linkages between two d-Glc*p*NAc residues (A and D) of the K102 and K47 CPSs of *A. baumannii* KZ-1102 and NIPH601 with the production of monomers and dimers of the K102 and K47 units, respectively. Considering that the structures of K102 and K47 CPS are similar and that the linkages between the K102 and K47 units that are cleaved by Cato_gp43 and TaPaz_gp79 are the same, it can be assumed that the depolymerases are specific to both K102 and K47 CPSs.

The study of new CPSs produced by *A. baumannii* help to expand our understanding of the structure of the bacterial population of this microorganism and the diversity of K types circulating worldwide. This is also the basis for the selection and design of specific antibacterial agents targeting certain bacterial surface components.

## 4. Materials and Methods

### 4.1. Bacterial Isolate and Antimicrobial Susceptibility

*A. baumannii* KZ-1102 was isolated from a sputum specimen in April 2017 in Astana, Kazakhstan. The isolate was referred to the national sentinel surveillance program of antimicrobial resistance in nosocomial bacterial pathogens conducted by the Institute of Antimicrobial Chemotherapy, Smolensk State Medical University (Smolensk, Russia) for further characterization and then deposited to the State Collection of Pathogenic Microorganisms and Cell Cultures (SCPM-Obolensk) under accession number B-22387. *A. baumannii* KZ-1102 was tested for susceptibility to antibiotics using the reference broth-microdilution susceptibility testing method [17] according to the EUCAST standards [18].

### 4.2. Sequencing and Bioinformatic Analysis

The whole-genome sequence of the KZ-1102 isolate was obtained on a MiSeq platform using a Nextera DNA library preparation kit (Illumina, San Diego, CA, USA). The short-read sequence data were assembled using SPAdes v. 3.13 [29]. MLST was performed by submitting the genome assembly to the PubMLST database [30] available at https://pubmlst.org/organisms/acinetobacter-baumannii (accessed on 12 March 2025). The K locus sequence was extracted and then subjected to KL typing using the Kaptive search tool [12,31]. The KZ-1102 genome region containing the fully annotated sequence of KL102 has been deposited to GenBank under accession number MK399429. A comparative analysis of KL sequences was performed using Clinker [23]. The analysis of the amino acid sequence of depolymerase Cato_gp43 was performed using the HHpred search (PDB70_mmCIF70_30_Mar and PfamA-v37 databases, HHpred probability > 95%) [26], and the BLASTp [32].

### 4.3. Isolation of K102 CPS

Bacteria were cultivated in 2TY (16 g Bacto tryptone, 10 g Bacto yeast extract, and 5 g NaCl) media overnight at 37 °C. Cells were harvested by centrifugation (10,000× *g*, 20 min), washed with phosphate-buffered saline, suspended in aqueous 70% acetone, precipitated, and dried.

Bacterial cells (2.02 g) were extracted with phenol-water [24]; the extract was dialyzed without layer separation and freed from insoluble contaminations by centrifugation at 12,000× *g* for 20 min. The resultant solution was treated with cold aqueous 50% CCl_3_CO_2_H at 0 °C; after centrifugation, the supernatant was dialyzed against distilled water, concentrated, and applied to an XK 26 mm (width) × 70 cm (height) column (gel layer, 560 mm) (GE Healthcare Life Sciences, Chicago, IL, USA) of Sephadex G-50 Superfine (Amersham Biosciences, Uppsala, Sweden). Elution with 0.1% acetic acid was monitored using a UV detector (Uvicord, Sweden) at 206 nm to obtain a purified CPS sample (240 mg). The CPS sample (100 mg) was hydrolyzed with 2% CH_3_CO_2_H (100 °C, 2 h). Fractionation of the products by gel-permeation chromatography on a column (56 × 2.5 cm) of Sephadex G-50 Superfine (GE Healthcare Life Sciences, Chicago, IL, USA) in 0.05 M pyridinium acetate buffer (pH 4.5) as eluent gave a purified CPS sample (26.8 mg).

### 4.4. Sugar Analysis

The CPS sample (1 mg) was hydrolyzed with 2 M CF_3_CO_2_H (120 °C, 2 h). Monosaccharides were analyzed by GLC of the alditol acetates on a Maestro chromatograph (Interlab, Moscow, Russia) equipped with an HP-5 column (0.32 mm × 30 m) using a temperature program of 160 °C (1 min) to 290 °C at 7 °C min^−1^.

### 4.5. Smith Degradation

CPS (16.3 mg) was oxidized in the dark with 1% NaIO_4_ (20 °C, 48 h), and 40 mg NaBH_4_ was added. After 16 h, the solution was acidified with concentrated acetic acid and evaporated. Boric acid was removed by evaporation with methanol and acetic acid (four times), and the modified CPS (11.6 mg) was isolated by GPC on a Sephadex G-50 column. Following hydrolysis with 2% acetic acid (100 °C, 2 h), the GPC of the products on a Sephadex G-25 column (1.2 × 110 cm) in water gave trisaccharide (2.3 mg).

### 4.6. Preparation of Recombinant Depolymerase Cato_Gp43 for CPS-Depolymerization

The expression vector pTSL [33], which contains the fragment of the gene corresponding to the phage-derived depolymerase Cato_gp43 lacking the N-terminal domain [15], was transformed into chemically competent *Escherichia coli* BL21 (DE3) cells. Protein expression was performed in a 2TY medium supplemented with ampicillin at 200 µg/mL. Transformed cells were grown at 37 °C until the optical density reached 0.7 at 600 nm. The medium was cooled to 18 °C, followed by expression induction by the addition of isopropyl-1-thio-β-d-galactopyranoside (IPTG) to a final concentration of 0.5 mM. After incubation for 16 h at 18 °C, the cells were harvested by centrifugation at 4000× *g* for 20 min at 4 °C. The cell pellets were then resuspended in buffer A (20 mM Tris pH 8.0, 200 mM NaCl) and sonicated. The lysates were cleared via centrifugation at 15,000× *g* for 15 min and then loaded into 5-mL Ni^2+^-charged GE HisTrap columns (GE Healthcare Life Sciences, Chicago, IL, USA) equilibrated with buffer A. The proteins were eluted using a 50–200 mM imidazole step gradient in buffer A. His-tag and SlyD digestion was achieved by incubation with tobacco etch virus (TEV) protease at a protease/protein ratio of 1/100 (wt/wt) overnight with simultaneous dialysis in 20 mM Tris-HCl buffer (pH 8.0) containing 1.0 mM 2-mercaptoethanol at room temperature. The cleaved protein was clarified by filtration and applied to an ion-exchange MonoQ 10/100 GL column (GE Healthcare Life Sciences, Chicago, IL, USA). The protein concentration was determined using the Bradford method with bovine serum albumin (BSA) as the standard.

### 4.7. Depolymerization of K102 CPS by Recombinant Depolymerase

Purified K102 CPS was solubilized in 20 mM Tris-HCl (pH 8.0) buffer, and 300 μg of recombinant depolymerase was added for digestion. The reaction mixture was then incubated at 37 °C. CPS digestion products were fractionated by gel permeation chromatography on an XK 16 mm × 100 cm column (gel layer, 800 mm) (GE Healthcare Life Sciences, Chicago, IL, USA) of Fractogel TSK HW-40S (Toyo Soda, Tokyo, Japan) in 1% acetic acid.

### 4.8. NMR Spectroscopy

A sample of purified K102 CPS was deuterium-exchanged by freeze-drying from 99.9% D_2_O and then examined as a solution in 99.95% D_2_O. NMR spectra were recorded on a Bruker Avance II 600 MHz spectrometer (Bremen, Germany) at 60 °C. Sodium 3-trimethylsilylpropanoate-2,2,3,3-d4 (δ_H_ 0, δ_C_ −1.6) was used as an internal reference for calibration. Two-dimensional NMR spectra were obtained using Bruker TopSpin 2.1 program, and the Bruker TopSpin 3.6.60 was used to acquire and process the NMR data. A 60-ms MLEV-17 spin-lock time and a 150-ms mixing time were used in the ^1^H-^1^H TOCSY and ROESY experiments, respectively.

### 4.9. Mass Spectrometry

High-resolution electrospray ionization (HR ESI) mass spectrometry was performed in the positive ion mode using a micrOTOF II instrument (Bruker Daltonics, Bremen, Germany). Oligosaccharide samples (~50 ng L^−1^) were dissolved in a 1:1 (*v*/*v*) water–acetonitrile mixture and injected using a syringe at a flow rate of 3 μL min^−1^. The capillary entrance voltage was set at 3200 V, and the interface temperature was set at 180 °C. Nitrogen was used as the drying gas. Mass range was from *m*/*z* 50 to 3500 Da. Internal calibration was performed using ESI Calibrant Solution (Agilent, Santa Clara, CA, USA).

## Figures and Tables

**Table 1:** Chemical shifts ^1^H and ^13^C NMR of the K102 CPS of *A. baumannii* KZ-1102.

Sugar Residue	C-1 *H-1*	C-2 *H-2*	C-3 *H-3*	C-4 *H-4*	C-5 *H-5*	C-6 *H-6 (6a,6b)*
CPS *A. baumannii* K102						
→6)-α-d-Glc*p*NAc-(1→	98.3	55.0	72.6	70.9	71.9	66.2
**A**	*4.94*	*3.95*	*3.81*	*3.69*	*3.80*	*3.71*, *4.08*
→3)-α-d-Glc*p*NAc-(1→	98.3	53.2	78.4	72.1	73.2	61.6
**B**	*4.93*	*4.12*	*3.97*	*3.78*	*3.77*	*3.81*, *3.86*
→3,4)-α-d-Gal*p*-(1→	100.1	69.2	80.0	77.3	72.9	60.7
**C**	*5.53*	*4.08*	*3.90*	*4.31*	*3.98*	*3.67*, *3.69*
→6)-α-d-Glc*p*NAc-(1→	98.7	55.2	71.9	70.9	71.9	66.4
**D**	*4.96*	*3.92*	*3.88*	*3.69*	*4.34*	*3.71*, *4.08*
β-d-Glc*p*-(1→	106.0	75.0	77.0	71.3	77.3	62.2
**E**	*4.60*	*3.17*	*3.51*	*3.36*	*3.44*	*3.69*, *3.89*

^1^H NMR chemical shifts are Italicized. Chemical shifts for the *N*-acetyl group of CPS are at δ_H_ 2.09, δ_C_ 23.8–23.7 (Me), and 175.7 (CO).

**Table 2:** Chemical shifts ^1^H and ^13^C NMR of OS1 derived after Smith degradation of the K102 CPS of *A. baumannii* KZ-1102.

Sugar Residue	C-1 *H-1*	C-2 *H-2*	C-3 *H-3*	C-4 *H-4*	C-5 *H-5*	C-6 *H-6 (6a,6b)*
OS1 After Smith Degradation						
α-d-Gal*p*-(1→	100.4	69.8	70.6	70.3	71.7	61.9
**C**	*5.43*	*3.83*	*3.78*	*3.99*	*3.95*	*3.75*, *3.75*
→3)-α- d-Glc*p*NAc-(1→	98.5	53.2	78.7	72.1	73.2	61.6
**B**	*4.87*	*4.09*	*3.94*	*3.75*	*3.72*	*3.87*, *3.79*
→1)-Gro	69.8	72.1	63.8			
**A’**	*3.58*, *3.76*	*3.88*	*3.69*, *3.65*			

^1^H NMR chemical shifts are italicized. Chemical shifts for the *N*-acetyl group of OS1 at δ_H_ 2.06, δ_C_ 23.3–23.2 (Me), and 175.4 (CO).

**Table 3:** Chemical shifts ^1^H and ^13^C NMR spectra of OS2 after depolymerization by Cato_gp43.

Sugar Residue	C-1 *H-1*	C-2 *H-2*	C-3 *H-3*	C-4 *H-4*	C-5 *H-5*	C-6 *H-6 (6a,6b)*
OS2						
→6)-α-d-Glc*p*NAc-(1→	98.6	55.2	72.2	71.3	73.3	61.9
**A**	*4.92*	*3.90*	*3.79*	*3.51*	*3.72*	*3.81*, *3.81*
→3)-α-d-Glc*p*NAc	92.5	53.7	78.1	72.8	73.3	60.7
**Bα**	*5.17*	*4.03*	*3.95*	*3.88*	*3.72*	*3.74*, *3.87*
→3)-β-d-Glc*p*NAc	96.0	56.5	80.2	72.3	73.3	60.7
**Bβ**	*4.76*	*3.77*	*3.76*	*3.78*	*3.72*	*3.74*, *3.87*
→3,4)-α-d-Gal*p*-(1→	100.1	69.2	79.9	77.2	72.9	61.8
**C**	*5.55*	*4.07*	*3.88*	*4.31*	*3.95*	*3.82*, *3.82*
→6)-α-d-Glc*p*NAc-(1→	98.8	55.2	72.3	70.7	71.9	65.9
**D**	*4.94*	*3.91*	*3.77*	*3.71*	*4.36*	*3.70*, *4.08*
β-d-Glc*p*-(1→	106.2	75.0	77.1	71.1	77.4	62.2
**E**	*4.58*	*3.17*	*3.49*	*3.39*	*3.41*	*3.71*, *3.89*

^1^H NMR chemical shifts are italicized. Chemical shifts for the N-acetyl group of OS2 at δ_H_ 2.07, δ_C_ 23.4–23.3 (Me), and 175.9 (CO).

## Data Availability

MK399429 (KL102 GenBank accession number).
